# Effects of exogenous retinoic acid on ocular parameters in Guinea pigs with form deprivation myopia

**DOI:** 10.3389/fcell.2023.1160897

**Published:** 2023-03-17

**Authors:** Yajun Wu, Yuliang Feng, Jiasong Yang, Hua Fan, Zitong Yu, Xiaolin Xie, Yumeng Dai, Xin Huang, Wensheng Li

**Affiliations:** ^1^ Aier School of Ophthalmology, Central South University, Changsha, Hunan, China; ^2^ Department of Ophthalmology, Shanghai Aier Ophthalmology Hospital, Shanghai, China; ^3^ Shanghai Aier Eye Institute, Shanghai, China; ^4^ Department of Ophthalmology, Jiangxi Provincial People’s Hospital, The First Affiliated Hospital of Nanchang Medical College, Nanchang, Jiangxi, China

**Keywords:** choroidal thickness, EDI-OCT, form deprivation myopia, Guinea pigs, myopia, retinal thickness, retinoic acid

## Abstract

**Aim:** Myopia is a common chronic eye disease, this study is to investigate the effects of exogenous retinoic acid (RA) on intraocular parameters, especially choroidal thickness (CT) and retinal thickness (RT), in guinea pigs with form deprivation myopia (FDM).

**Methods:** A total of 80 male guinea pigs were divided randomly into 4 groups: Control, FDM, FDM + RA, and FDM + Citral groups. The FDM + RA group was given 24 mg/kg RA dissolved in 0.4 mL peanut oil; the FDM + Citral group was given citral 445 mg/kg dissolved in 0.4 mL peanut oil; The other two groups were given 0.4 mL peanut oil. After 4 weeks, the refractive error (RE), axial length (AL), and intraocular pressure (IOP) of all guinea pigs were measured, and the parameters of RT and CT were obtained using enhanced depth imaging optical coherence tomography (EDI-OCT).

**Results:** After 4 weeks, both the RE and AL in the FDM and FDM + RA groups were increased, and the RT and CT in both groups were smaller than those in the Control group (*p* < 0.05). Only the IOP of the right eye in the FDM + RA group increased significantly (*p* < 0.05). The RT of the right eye of the 4 groups was compared: Control group > FDM + Citral group > FDM group > FDM + RA group. Compared with the RT of the left eye and the right eye among the 4 groups, the RT of the right eye in the FDM and FDM + RA groups was significantly less than that in the left eye (*p* < 0.05). Moreover, the CT of the right eye in the Control group was greater than that in the other three groups (*p* < 0.0001). There was no significant difference in the CT among the FDM, FDM + RA, and FDM + Citral groups (*p* > 0.05). In contrast to the RT results, the CT results of the left and right eyes in the FDM + Citral group showed statistically significant differences (*p* < 0.05).

**Conclusion:** RA participates in the progression of FDM as a regulatory factor. Exogenous RA can increase the RE, AL, and IOP of FDM guinea pigs, and might aggravate the retinal thinning of FDM guinea pigs. Citral can inhibit these changes, but RA might not affect the thickness of the choroid.

## Introduction

Myopia is the most common refractive error (RE) (Dolgin, 2015), and it's incidence is still on the rise (Han et al., 2022), reaching epidemic levels (Medina, 2021). The highest incidence of myopia in the world is in East Asia, where the rate among children alone is as high as 7%–30% (Lee et al., 2022), and the prevalence of myopia in young adults is as high as 80%–90%, among which the prevalence of high myopia (HM) [diopter (D) < −6 or axial length (AL) > 26 mm] is approximately 10%–20% (Morgan et al., 2018). Moreover, among East Asian countries, the prevalence of myopia among students is particularly severe in China, where it is predicted that, by 2050, the prevalence of myopia among children and adolescents aged 3–19 years old might be as high as 84% (Dong et al., 2020). In addition, the prevalence of myopia among high-school students in East and Southeast Asia is approximately 30-times higher than in sub-Saharan Africa (Jonas et al., 2021). The situation of myopia among adolescents is particularly serious, especially in East Asian countries. Adolescents (young people aged 10–19) (Das et al., 2017) are crucial to the future development and progress of a country, and adolescent eye health is related to the national health (Shi et al., 2022), therefore, the high incidence of myopia will bring a burden to society and families. Thus, to slow down or even prevent the high incidence rate of myopia, it is necessary to clarify the mechanism of its occurrence and development, and this is the main direction of myopia research at present.

Retinoic acid (RA) is an acid derivative of vitamin A, which is the one-way oxidation product of photosensitive cells in the eye (Brown et al., 2022a; Brown et al., 2022b), and RA has an important role in eye development. Citral is a competitive inhibitor of the key dehydrogenase required for RA generation. Studies have shown that the retinal RA content of myopia is significantly higher than that of non-myopia (Brown et al., 2022a; Brown et al., 2022b). Also, Wang et al. found that the level of RA and the expression level of Zonula occludens-1 (ZO-1) and occludin in the retinal pigment epithelium (RPE) –choroid complex of lens induced myopia (LIM) guinea pigs were significantly increased, whereas the expression of RA and the proteins of ZO-1and occludin in LIM guinea pigs treated with RA antagonists were inhibited (Wang et al., 2014). These studies show that RA is involved in myopia regulation. However, the specific role and mechanism of RA in myopia are not clear, as well as its effects on the retina and choroid.

Furthermore, to evaluate the severity of myopia, it is important to obtain relevant ophthalmic indicators. Generally, RE and AL are considered as the most important parameters, and almost all myopia studies will obtain these two parameters, or at least one of them (Tideman et al., 2018; Hughes et al., 2020), also, which will be used as the indicator to evaluate the prevention and control effect of myopia (Chamberlain et al., 2021). The appearance of imaging instruments has provided assistance for the acquisition of ophthalmic parameters. For example, intraocular lens-master (IOL-master), A-scan, and optical coherence tomography (OCT) can be used for the acquisition of AL, of which IOL-master is used widely in clinic, whereas A-scan is used widely in animal research of myopia (Yang et al., 2021). Enhanced depth imaging OCT (EDI-OCT) can be used to acquire choroidal images, compared with ordinary OCT, which focuses on the retina, and it can improve the quality of choroidal imaging significantly (Sodi et al., 2018a; Sodi et al., 2018b). Thus, EDI-OCT can be used to obtain clear images of the retina and choroid, and the built-in software of its detection system can measure retinal thickness (RT) and choroidal thickness (CT) manually. Moreover, many studies have proved that myopia can also affect RT and CT. Zhang et al. (Zhang et al., 2019) measured the CT of spontaneous myopia guinea pigs, form deprivation myopia (FDM) guinea pigs, and LIM guinea pigs, finding that the CT of the three groups decreased in the myopia stage, but increased in the myopia recovery stage, so they suggested that CT could be used as an early predictor of myopia. Also, Jonas et al. (Jonas et al., 2019) believed that axial myopia could lead to thinning of the retina at the posterior pole, especially around the optic disc, and that AL was related to the RT around the optic disc, but not to the RT of the macula. They believed that axial myopia would cause additional Bruch membrane to be produced in the area behind the equator of the eye, thus causing thinning of the retina around the optic disc, whereas the macular area would not be affected. However, the CT and RT measured in most studies are usually the macular part, not the periphery of the optic disc (Tian et al., 2021).

At present, although it has been known that RA might be involved in the occurrence and development of myopia in guinea pigs, its mechanism is not clear, and no studies have reported the effect of RA on RT and CT in FDM guinea pigs. Therefore, this study intends to explore the effect of RA on the intraocular parameters of guinea pigs with myopia, especially the effects of RA on peripheral RT and CT of the optic disc in FDM guinea pigs by using EDI-OCT.

## Materials and methods

### Animals

A total of 80 male 2-week-old British guinea pigs [Beijing Weitong Lihua Experimental Animal Technology Co., Ltd., China, production license number: SCXK (Beijing) 2021-0011] were selected. All guinea pigs were kept in a clean environment at a room temperature of 18–29 °C (daily temperature difference ≤4 °C) and relative humidity of 40–70%. Inclusion criteria: 1. male 2-week-old guinea pigs, of body weight 200–220g, and body weight individual value within ± 20% of the mean; 2. The cornea is clear and transparent, the fundus is normal, and there is no obvious anisometropia. Exclusion criteria: 1. Guinea pigs over 2 weeks old; 2. Guinea pigs whose weight is not within the standard range; 3. Guinea pigs with anisometropia >1D, amblyopia, and myopia; 4. There are abnormalities in the eyes, such as corneal trauma or vitreous opacity; 5. Guinea pigs in poor condition and unable to continue the experiment or were injured or died during the experiment.

The breeding environment conforms to the national standard GB14925-2010 of the People’s Republic of China. There was one cage for every three guinea pigs, with 12/12 h of light alternating between day and night (light: 07:70 AM to 17:30 PM), all animals drink and eat freely. The relevant contents and procedures involved in this test comply with the relevant provisions of the Institutional Animal Care and Use Committee (IACUC) and have been approved by the Ethics Committee of West China-Frontier Pharma Tech Co., Ltd., Chengdu, Sichuan, China. The ethics number is: IACUC- SW-S2022022023-P001-01.

### Animal grouping and model establishment

There were 4 groups: Control group, FDM group, FDM + RA group, and FDM + Citral group. Guinea pigs were divided randomly according to body weight, with 20 males per group.

The eyes of the control group were not treated. The right eyes of the guinea pigs in the FDM group, the FDM + RA group, and the FDM + Citral group were myopia, and the left eyes were self-control. The white non-toxic No.6 latex balloon is used to make the head cover, which covers the right eye of all FDM guinea pigs but does not compress the cornea and eyelid of the right eye, to ensure that the right eye can blink freely; at the same time, the left eye, mouth, nose, and ears are fully exposed. Wearing of the head cover was checked every 1 day, and any damaged, displaced, and tight head cover was replaced in time to ensure that the right eye of the guinea pig is covered continuously but can blink freely. After 4 weeks, the RE, IOP, and AL of all guinea pigs were measured, and the model was successful if the AL increased and RE decreased. The FDM + RA group was given RA by gavage (24 mg/kg dissolved in 0.4 mL peanut oil, all-trans, Sigma-Aldrich, United States); the FDM + Citral group was given citral by gavage (445 mg/kg dissolved in 0.4 mL peanut oil, mixture of cis and trans, Macklin, China) (Yu et al., 2021), starting at 10 a.m. each time, for 4 weeks, once every 3 days; the other two groups were given 0.4 mL peanut oil at the same time point.

### Retinography and measurement of AL and IOP

RE and AL of all guinea pigs were measured at the beginning of the experiment and 4 weeks later (Since these two data are the key to judge whether guinea pigs successfully induce relative myopia, we measured them before and after induction to determine the degree of myopia). The infrared band-light retinoscope (Suzhou 66 Vision Technology Co., Ltd., China) was used to obtain the RE of all guinea pigs. Compound topiricamine eye drops were used to dilate the eyes. After three times of dosing, the eyes were examined in a dark room 15 min later. The RE in horizontal and vertical positions were detected respectively by an experienced optometrist at the working distance of 50 cm and 0.25 D, and half of the astigmatism was included in the equivalent spherical lens (Luo et al., 2017). OD-1 (Kaixin, Xuzhou, China) small animal A scan was used to measure AL. Aubucaine hydrochloride eye drops (1–2 drops) were placed on the eye surface of all guinea pigs 3 times, each time at an interval of 5 min. The small animal measurement mode was used, and the probe was perpendicular to the pupil area and touched the cornea (avoid pressing), and the value of the waveform contraction in front of the retina was read as AL, accurate to 0.01 mm. The measurement was repeated 3 times, and the average value was taken. The IOP of guinea pigs was measured with TONOVET (TV02) tonometer (Icare, Finland). Each eye was measured 3 times and the average value was taken.

All guinea pigs were kept awake during the examination of RE, AL and IOP.

### Measurement of retina and choroid images by EDI-OCT

**FIGURE 1:**
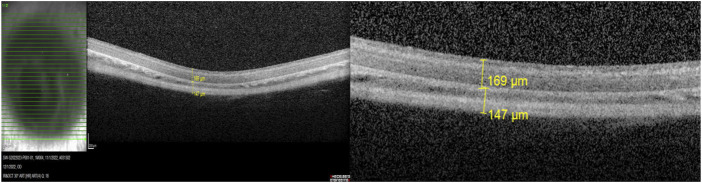
Structure of retina and choroid under EDI-OCT.

After 4 weeks, the CT and RT of all guinea pigs were measured using the EDI mode of OCT (Heidelberg, Germany). All guinea pigs were kept awake and their eyes were dilated with compound topicamide eye drops. After pacifying the guinea pigs, their whole body was wrapped, placed on the OCT examination table, and the eyes to be examined were exposed, so that the anteroposterior diameter of the eyes was consistent with the scanning indicator light source. Then, 31 layers below the optic disc from the bottom to the top was scanned, and the RT and CT of 1 disc diameter (DD) below the optic disc was measured using the manual measurement software provided by the detection system [RT is defined as the inner limiting membrane (ILM) of the retina to the retinal pigment epithelium (RPE), and the highly reflective band width outside the RPE is defined as CT (Sodi et al., 2018a; Sodi et al., 2018b)] (Figure 1).

### Statistical analysis

SPSS25.0 (IBM Corp., Armonk, NY, United States) was used for statistical analysis. All results were expressed as mean ± standard deviation (SD). The comparison of RE, AL, IOP, CT, and RT among the four groups was performed by one-way analysis of variance (ANOVA). The comparison between the left and right eyes among the groups was performed by paired *t*-test. *p* < 0.05 represents a difference with statistical significance.

## Results

### RE results of Guinea pigs in each group

**FIGURE 2:**
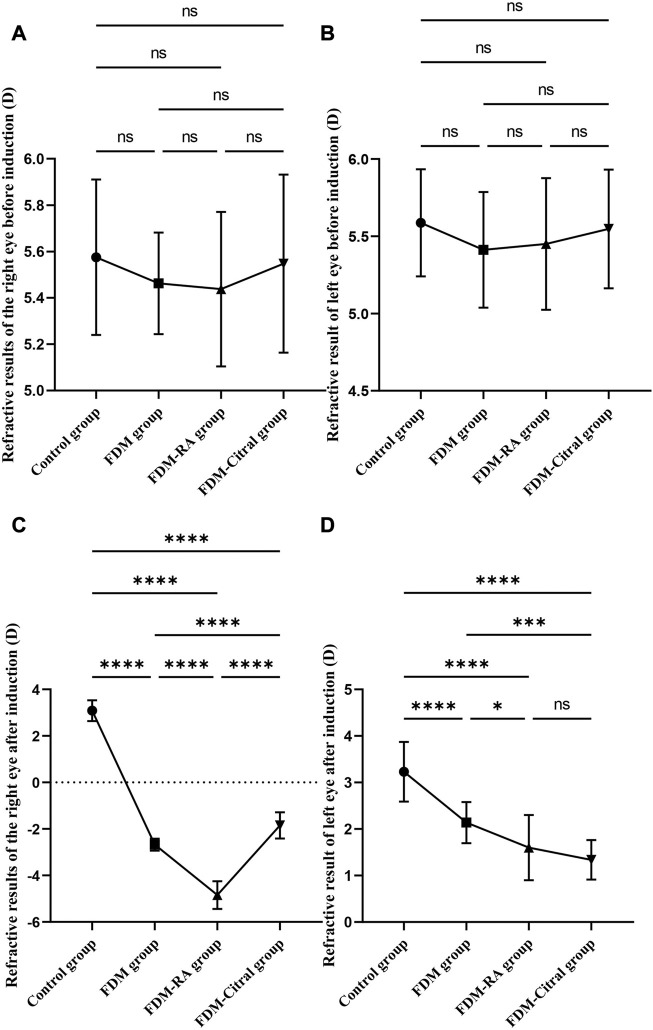
Comparative results of RE before and after induction in four groups (D). Note. **(A)**, Comparison of RE in the right eye before induction; **(B)**, Comparison of RE in the left eye before induction; **(C)**, Comparison of RE in the right eye after induction; **(D)**, Comparison of RE in the left eye after induction; **(D)**, diopter; ns means that the difference is not statistically significant; *Indicates *p* ≤ 0.05; ** Indicates *p* ≤ 0.01; *** Indicates *p* ≤ 0.001; **** Indicates *p* ≤ 0.0001.

There was no statistical significance in the comparison of RE between the four groups before induction (Figures 2A, B). After 4 weeks of induction, the comparison difference of RE in the right eye of each group was statistically significant, and the FDM + RA group had the smallest RE (Figure 2C). In the left eye, the comparison difference among other groups was statistically significant except that between FDM group and FDM + Citral group (Figure 2D).

**FIGURE 3:**
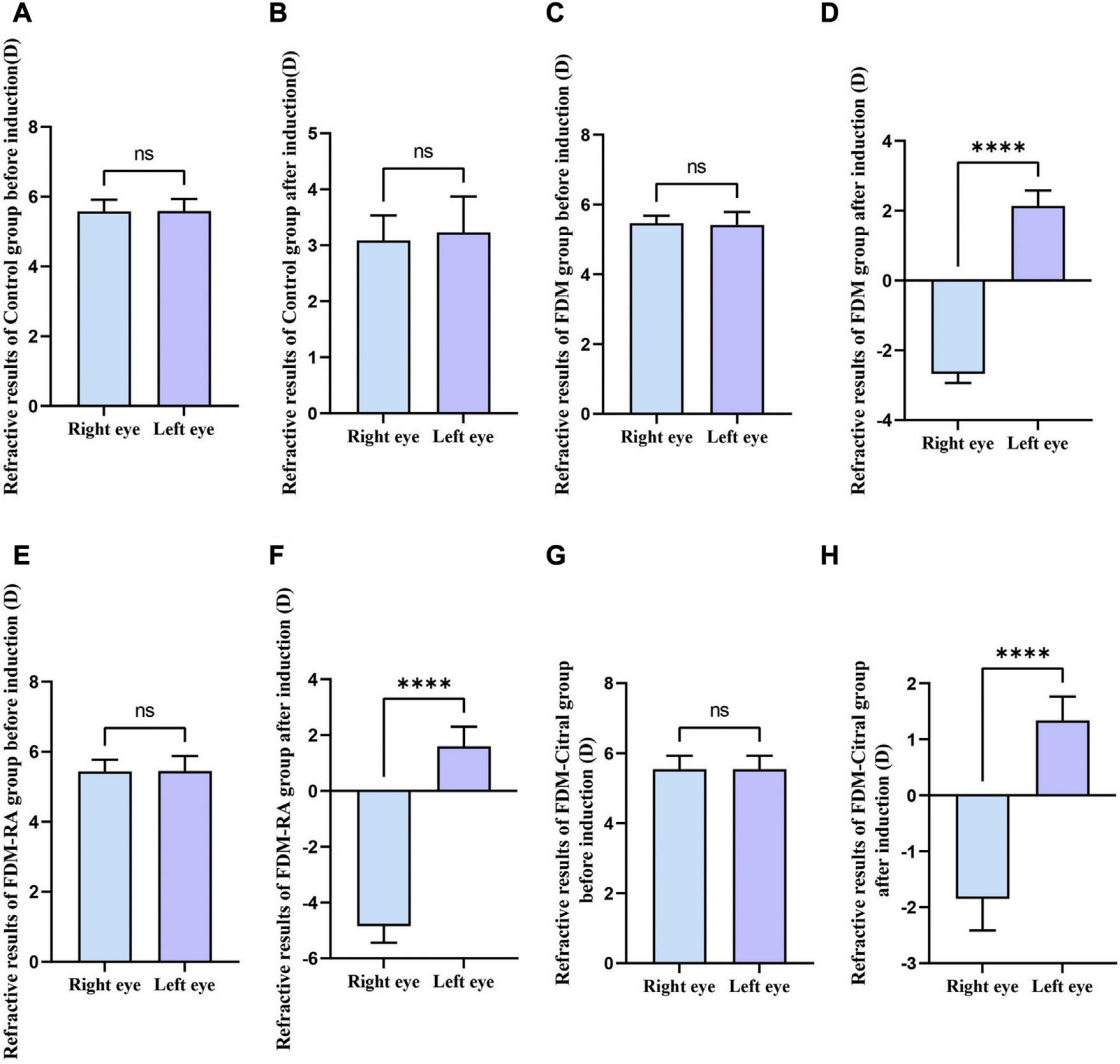
Comparison of RE between the left and right eyes before and after induction in each group (D). Note. **(A)**, Comparison of RE between left and right eyes before induction in Control group; **(B)**, Comparison of RE between left and right eyes after induction in Control group; **(C)**, Comparison of RE between left and right eyes before induction in FDM group; **(D)**, Comparison of RE between left and right eyes after induction in FDM group; **(E)**, Comparison of RE between left and right eyes before induction in FDM + RA group; **(F)**, Comparison of RE between left and right eyes after induction in FDM + RA group; **(G)**, Comparison of RE between left and right eyes before induction in FDM + Citral group; **(H)**, Comparison of RE between left and right eyes after induction in FDM + Citral group; **(D)**, diopter; ns means that the difference is not statistically significant; **** Indicates *p* ≤ 0.0001.

There was no statistically significant difference in RE between the left and right eyes of each group before induction (Figures 3A, C, E, G); The RE in the right eye of the Control group changed from 5.58 ± 0.34 to 3.09 ± 0.45 D, the difference of RE between the left and right eyes was also not statistically significant (Figure 3B); The RE of the right eye of FDM group changed from 5.46 ± 0.22 D to −2.68 ± 0.26 D; The RE of the right eye of FDM + RA group changed from 5.44 ± 0.33 D to −4.84 ± 0.59 D; The RE of the right eye of FDM + Citral group changed from 5.55 ± 0.38 D to −1.85 ± 0.56 D. There was statistically significant difference in RE between the left and right eyes in FDM group (Figure 3D), FDM + RA group (Figure 3F) and FDM + Citral group (Figure 3H). All details are shown in Table 1.

**TABLE 1:** RE of left and right eyes of guinea pigs before and after experimental induction (D).

Group (*n* = 80)	Before induced			T	P	After induced			T	P
	L	R	L-R			L	R	L-R		
Control group	5.59 ± 0.35	5.58 ± 0.34	0.01 ± 0.11	0.11	0.9084	3.23 ± 0.64	3.09 ± 0.45	0.15 ± 0.17	0.83	0.4119
FDM group	5.41 ± 0.37	5.46 ± 0.22	−0.05 ± 0.1	0.52	0.6089	2.14 ± 0.44	−2.68 ± 0.26	4.81 ± 0.11	42.18	<0.0001
FDM + RA group	5.45 ± 0.43	5.44 ± 0.33	0.01 ± 0.12	0.10	0.9182	1.60 ± 0.70	−4.84 ± 0.59	6.44 ± 0.21	31.39	<0.0001
FDM + Citral group	5.55 ± 0.38	5.55 ± 0.38	0.00 ± 0.12	0.00	>0.9999	1.34 ± 0.42	−1.85 ± 0.56	3.19 ± 0.16	20.20	<0.0001
F	0.91	0.83				44.16	958.90			
P	0.4384	0.4796				<0.0001	<0.0001			

Note: R, right eye; L, left eye; D, diopter; RE, refractive error; F, *f* value; P, *p* value; T, *t* value; *p* < 0.05 means statistically significant difference.

### AL results of Guinea pigs in each group

**FIGURE 4:**
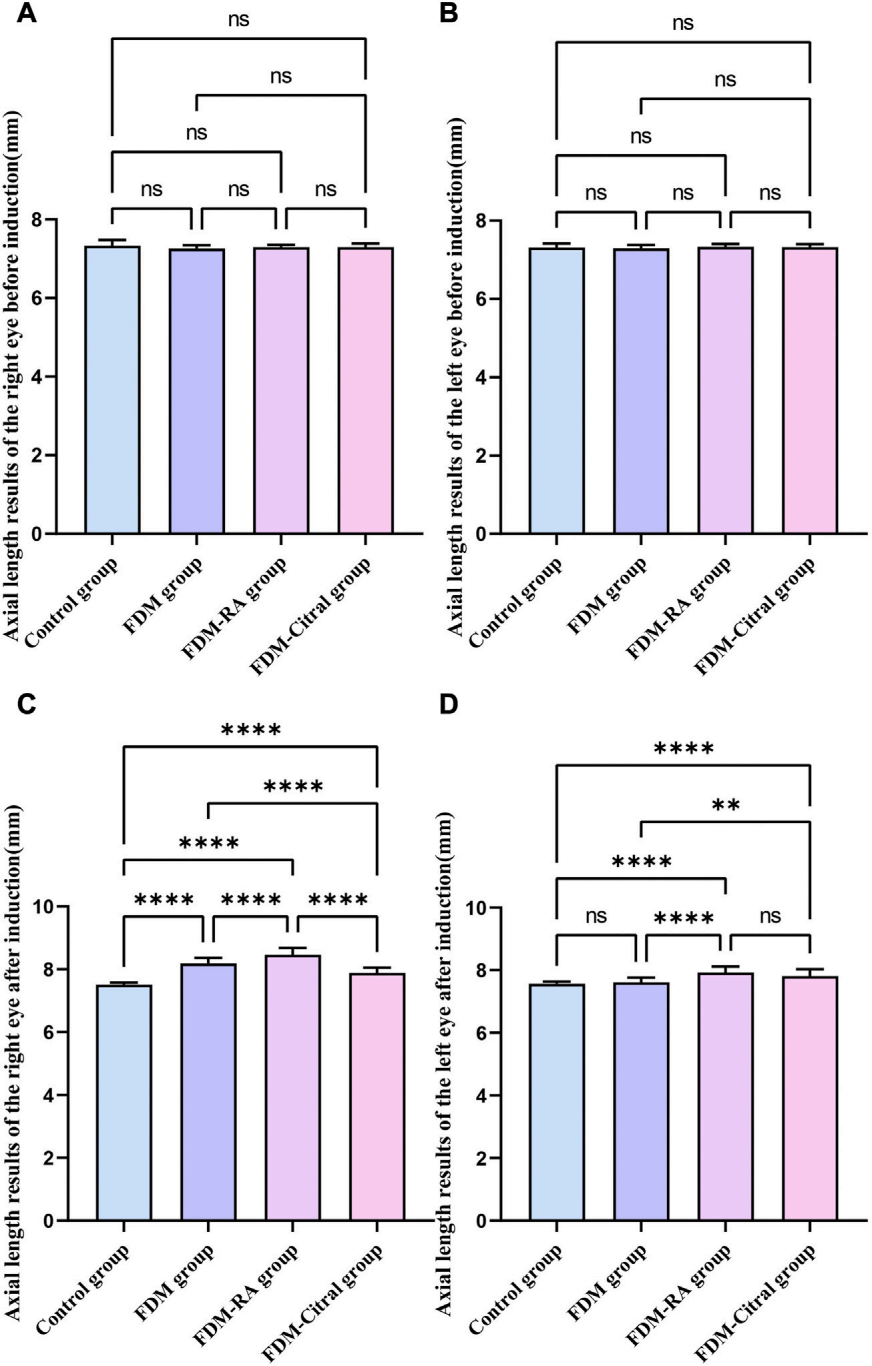
Comparison of AL results of four groups of guinea pigs (mm). Note. **(A)**, Comparison of AL in the right eye before induction; **(B)**, Comparison of AL in the left eye before induction; **(C)**, Comparison of AL in the right eye after induction; **(D)**, Comparison of AL in the left eye after induction; **(D)**, diopter; ns means that the difference is not statistically significant; *Indicates *p* ≤ 0.05; ** Indicates *p* ≤ 0.01; *** Indicates *p* ≤ 0.001; **** Indicates *p* ≤ 0.0001.

There was no statistical difference in the comparison of bilateral AL between the four groups before induction (Figures 4A, B); After induction, the difference of AL in the right eye between the four groups was statistically significant, and the AL in the FDM + RA group was the largest (Figure 4C); The comparison of left eye AL between the four groups after induction was statistically significant except for the Control group VS. FDM and FDM + RA group VS. FDM + Citral group (Figure 4D).

**FIGURE 5:**
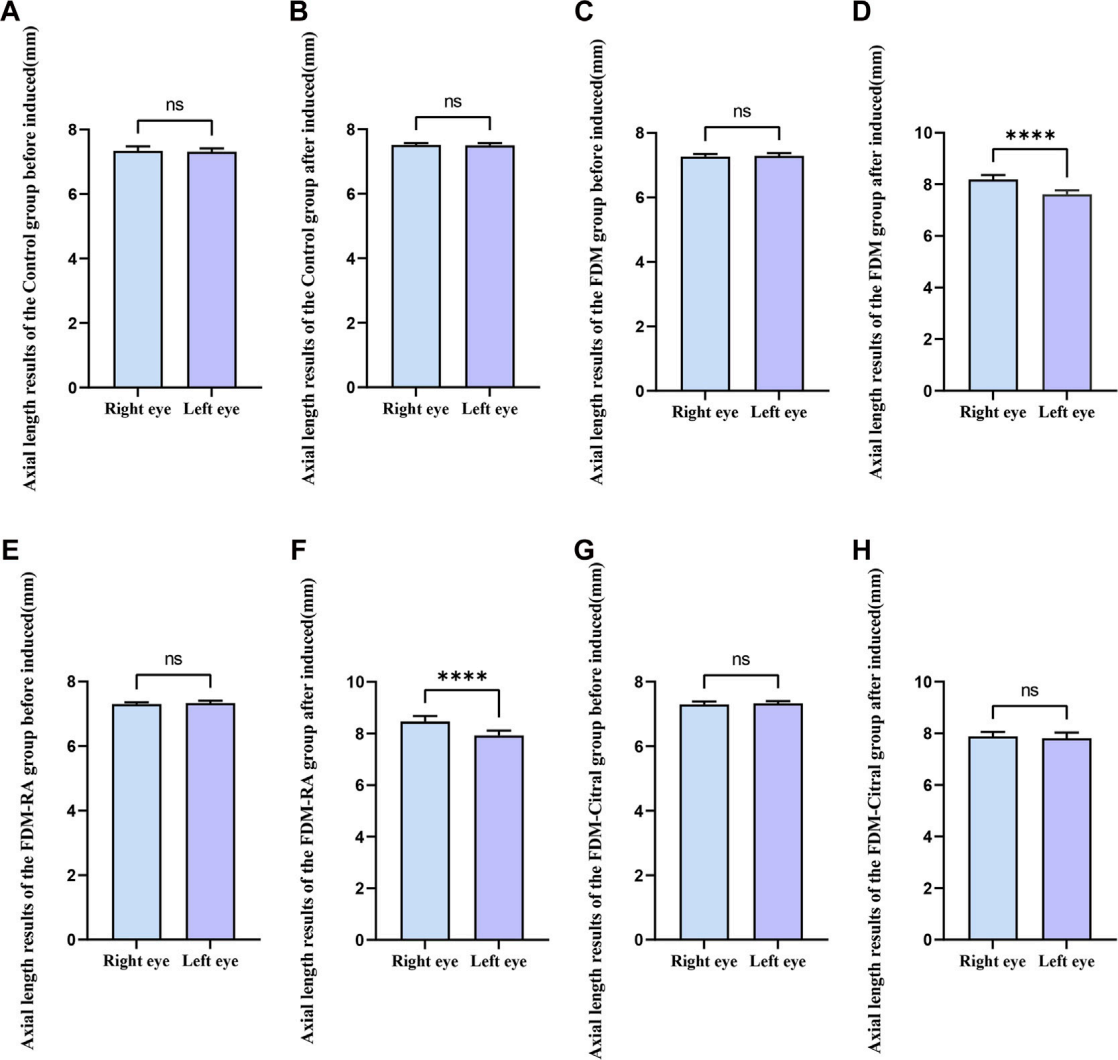
Comparison of AL between the left and right eyes in each group (mm). Note. **(A)**, Comparison of AL between left and right eyes before induction in Control group; **(B)**, Comparison of AL between left and right eyes after induction in Control group; **(C)**, Comparison of AL between left and right eyes before induction in FDM group; **(D)**, Comparison of AL between left and right eyes after induction in FDM group; **(E)**, Comparison of AL between left and right eyes before induction in FDM + RA group; **(F)**, Comparison of AL between left and right eyes after induction in FDM + RA group; **(G)**, Comparison of AL between left and right eyes before induction in FDM + Citral group; **(H)**, Comparison of AL between left and right eyes after induction in FDM + Citral group; AL, axial length; ns means that the difference is not statistically significant; **** Indicates *p* ≤ 0.0001.

Before induction, there was no statistical difference in AL between the left and right eyes in each group (Figures 5A, C, E, G); After induction, only the FDM group and FDM + RA group had statistically significant differences in AL between the left and right eyes (Figures 5D, F); There was no statistical difference in AL between the left and right eyes of the Control group and FDM + Citral group (Figures 5B, H); The changes in AL for each group are detailed in Table 2.

**TABLE 2:** AL of left and right eyes of guinea pigs before and after experimental induction (mm).

Group (*n* = 80)	Before induced			T	P	After induced			T	P
	L	R	L-R			L	R	L-R		
Control group	7.31 ± 0.10	7.33 ± 0.15	−0.02 ± 0.04	0.51	0.6154	7.56 ± 0.07	7.58 ± 0.06	−0.01 ± 0.02	0.65	0.5181
FDM group	7.29 ± 0.08	7.26 ± 0.08	0.03 ± 0.03	1.22	0.2304	7.62 ± 0.15	8.19 ± 0.18	−0.57 ± 0.05	11.46	<0.0001
FDM + RA group	7.33 ± 0.07	7.30 ± 0.05	0.03 ± 0.02	1.41	0.1654	7.93 ± 0.19	8.46 ± 0.21	−0.54 ± 0.06	8.41	<0.0001
FDM + Citral group	7.33 ± 0.07	7.30 ± 0.09	0.03 ± 0.03	1.17	0.2510	7.81 ± 0.22	7.88 ± 0.17	−0.07 ± 0.06	1.16	0.2530
F	0.86	1.84				20.94	106.10			
P	0.4678	0.1468				<0.0001	<0.0001			

Note: R, right eye; L, left eye; AL, axial length; FDM, form deprivation myopia; RA, retinoic acid; F, *f* value; P, *p* value; T, *t* value; *p* < 0.05 represents statistically significant difference.

### The result of IOP after induction

**FIGURE 6:**
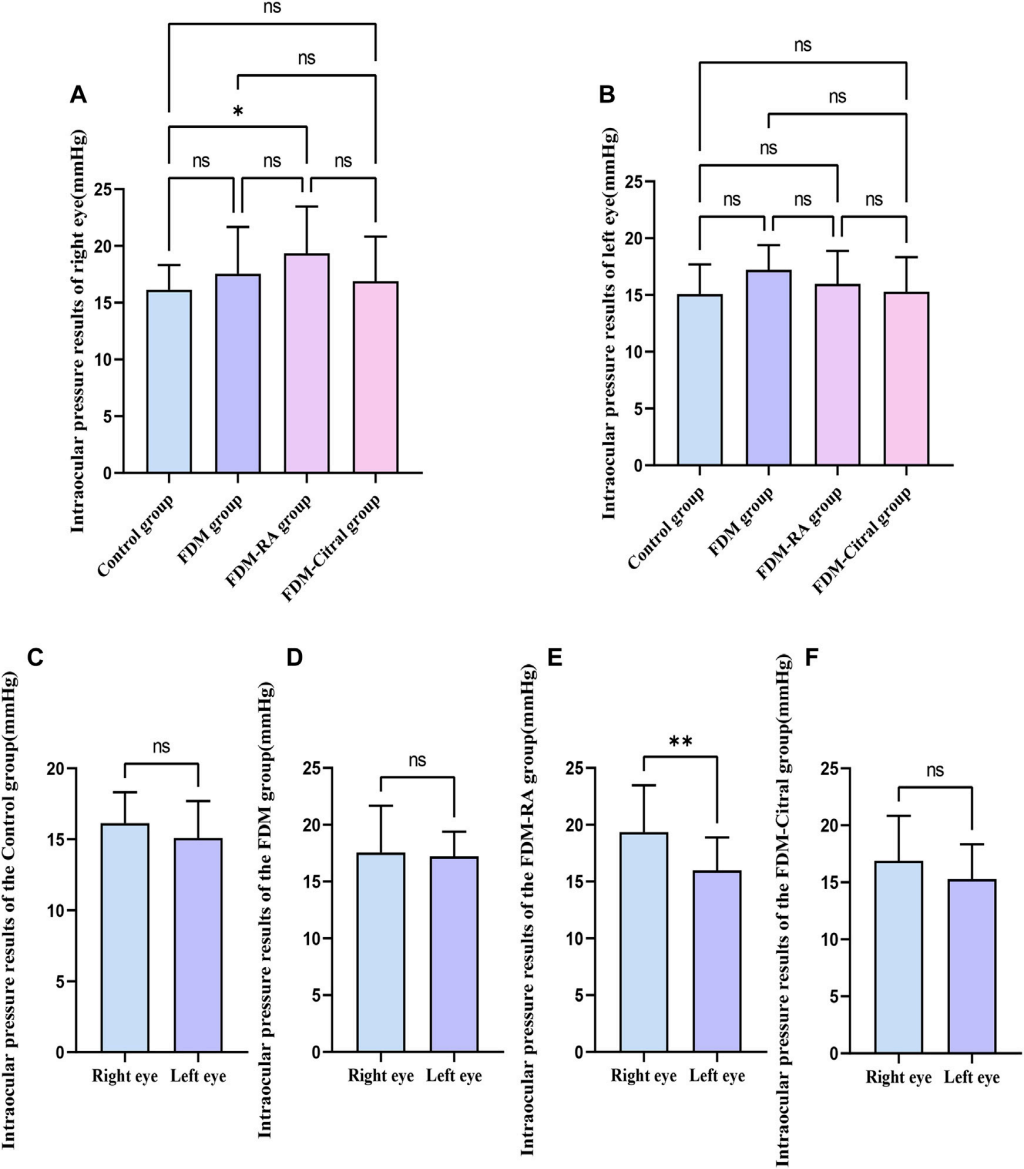
Comparison of IOP in each group (mmHg). Note. **(A)**, Comparison of IOP in the right eye; **(B)**, Comparison of IOP in the leftt eye; **(C)**, Comparison of IOP between left and right eyes in Control group; **(D)**, Comparison of IOP between left and right eyes in FDM group; **(E)**, Comparison of IOP between left and right eyes in FDM + RA group; **(F)**, Comparison of IOP between left and right eyes in FDM + Citral group; IOP, intraocular pressure; ns means that the difference is not statistically significant; *Indicates *p* ≤ 0.05; ** Indicates *p* ≤ 0.01.

The IOP of the right eye in the FDM + RA group was higher than that in the Control group (*p* ≤ 0.05), and the difference between the IOP of the left and right eyes in the FDM-RA group was also statistically significant (*p* ≤ 0.01). The IOP of the right eye was greater than that of the left eye, and there was no statistically significant difference between the groups and between the left and right eyes in each group. See Figure 6; Table 3 for details.

**TABLE 3:** IOP results of left and right eyes of guinea pigs in each group (mmHg).

Group (*n* = 80)	IOP			T	P
	L	R	L-R		
Control group	15.08 ± 2.60	16.13 ± 2.17	−1.05 ± 0.76	1.38	0.1747
FDM group	17.21 ± 2.18	17.56 ± 4.10	−0.35 ± 0.99	0.35	0.7271
FDM + RA group	15.98 ± 2.90	19.35 ± 4.13	−3.37 ± 1.13	2.99	0.0049
FDM + Citral group	15.29 ± 3.04	16.90 ± 3.93	−1.61 ± 1.04	1.55	0.1277
F	2.73	2.79			
P	0.0491	0.0459			

Note: R, right eye; L, left eye; IOP, intraocular pressure; FDM, form deprivation myopia; RA, retinoic acid; F, *f* value; P, *p* value; T, *t* value; *p* < 0.05 represents statistically significant difference.

### CT and RT results of Guinea pigs measured by EDI-OCT

**FIGURE 7:**
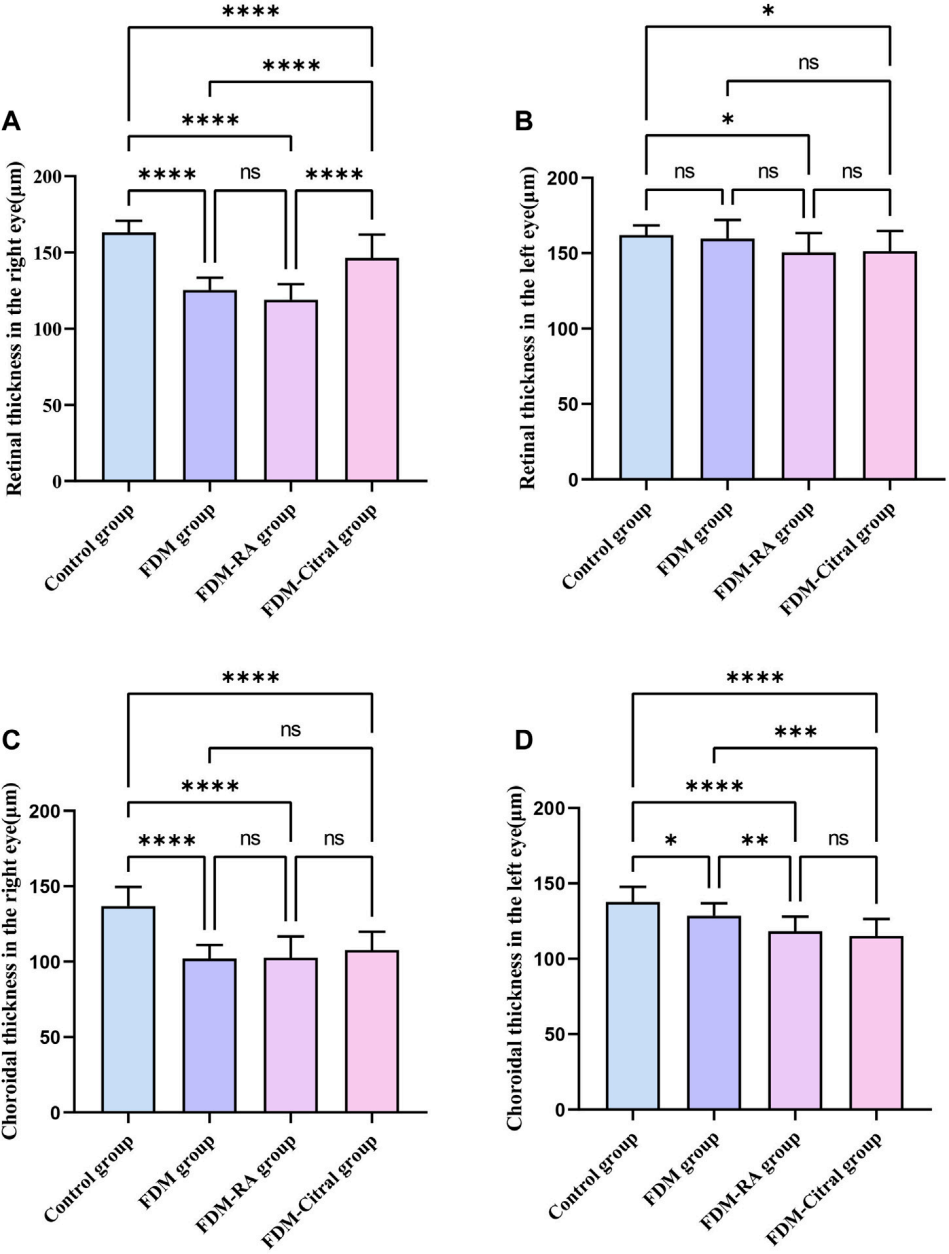
Comparative results of CT and RT in each group (μm). Note. **(A)**, Comparison of RT of right eye; **(B)**, Comparison of RT of left eye; **(C)**, Comparison of CT of right eye; **(D)**, Comparison of CT of left eye; ns means that the difference is not statistically significant; *Indicates *p* ≤ 0.05; ** Indicates *p* ≤ 0.01; *** Indicates *p* ≤ 0.001; **** Indicates *p* ≤ 0.0001.

Compared with RT in the right eye of the 4 groups, the Control group > FDM + Citral group > FDM group > FDM + RA group, there was no statistical significance between the FDM group and the FDM + RA group, and the comparison between the other two groups was statistically significant *p* < 0.0001 (Figure 7A). Compared with RT in the left eye of the 4 groups, only the Control group VS. FDM + Citral group and the Control group VS. FDM + RA group showed statistically significant differences (*p* < 0.05), while pairwise comparison among other groups showed no statistically significant differences (Figure 7B). Compared with the CT of the right eye of the 4 groups, the CT of the control group was larger than that of the other three groups, and the difference was statistically significant (*p* < 0.0001), there was no statistical difference between the FDM + Citral group, the FDM group, and the FDM + RA group (Figure 7C); There was no statistically significant difference between the FDM + RA VS. FDM + Citral groups compared with the CT of the left eye in the four groups, and the differences between the other groups were statistically significant (Figure 7D). Detailed data are shown in Tables 4, 5.

**TABLE 4:** CT results of left and right eyes of guinea pigs in each group measured by EDI-OCT (μm).

Group (*n* = 80)	CT			T	P
	L	R	L-R		
Control group	137.70 ± 9.92	136.90 ± 12.61	0.85 ± 3.59	0.24	0.8140
FDM group	128.60 ± 8.23	102.10 ± 9.01	26.48 ± 2.67	9.94	<0.0001
FDM + RA group	118.20 ± 9.70	102.60 ± 14.10	15.65 ± 3.83	4.09	0.0002
FDM + Citral group	115.20 ± 11.23	107.60 ± 12.13	7.57 ± 3.45	2.20	0.0335
F	22.69	38.05			
P	<0.0001	<0.0001			

Note: R, right eye; L, left eye; FDM, form deprivation myopia; RA, retinoic acid; CT, choroidal thickness; EDI-OCT, enhanced depth imaging-optical coherence tomography; F, *f* value; P, *p* value; T, *t* value; *p* < 0.05 represents statistically significant difference.

**TABLE 5:** RT results of left and right eyes of guinea pigs in each group measured by EDI-OCT (μm).

Group (*n* = 80)	RT			T	P
	L	R	L-R		
Control group	162.00 ± 6.41	163.20 ± 7.67	−1.15 ± 2.24	0.51	0.6099
FDM group	159.7 ± 12.39	125.30 ± 8.13	34.38 ± 3.24	10.63	<0.0001
FDM + RA group	150.60 ± 12.76	118.90 ± 10.30	31.66 ± 3.73	8.50	<0.0001
FDM + Citral group	151.40 ± 13.28	146.50 ± 15.29	4.91 ± 4.22	1.16	0.2509
F	5.15	68.09			
P	0.0026	<0.0001			

Note: R, right eye; L, left eye; FDM, form deprivation myopia; RA, retinoic acid; RT, retinal thickness; EDI-OCT, enhanced depth imaging-optical coherence tomography; F, *f* value; P, *p* value; T, *t* value; *p* < 0.05 represents statistically significant difference.

**FIGURE 8:**
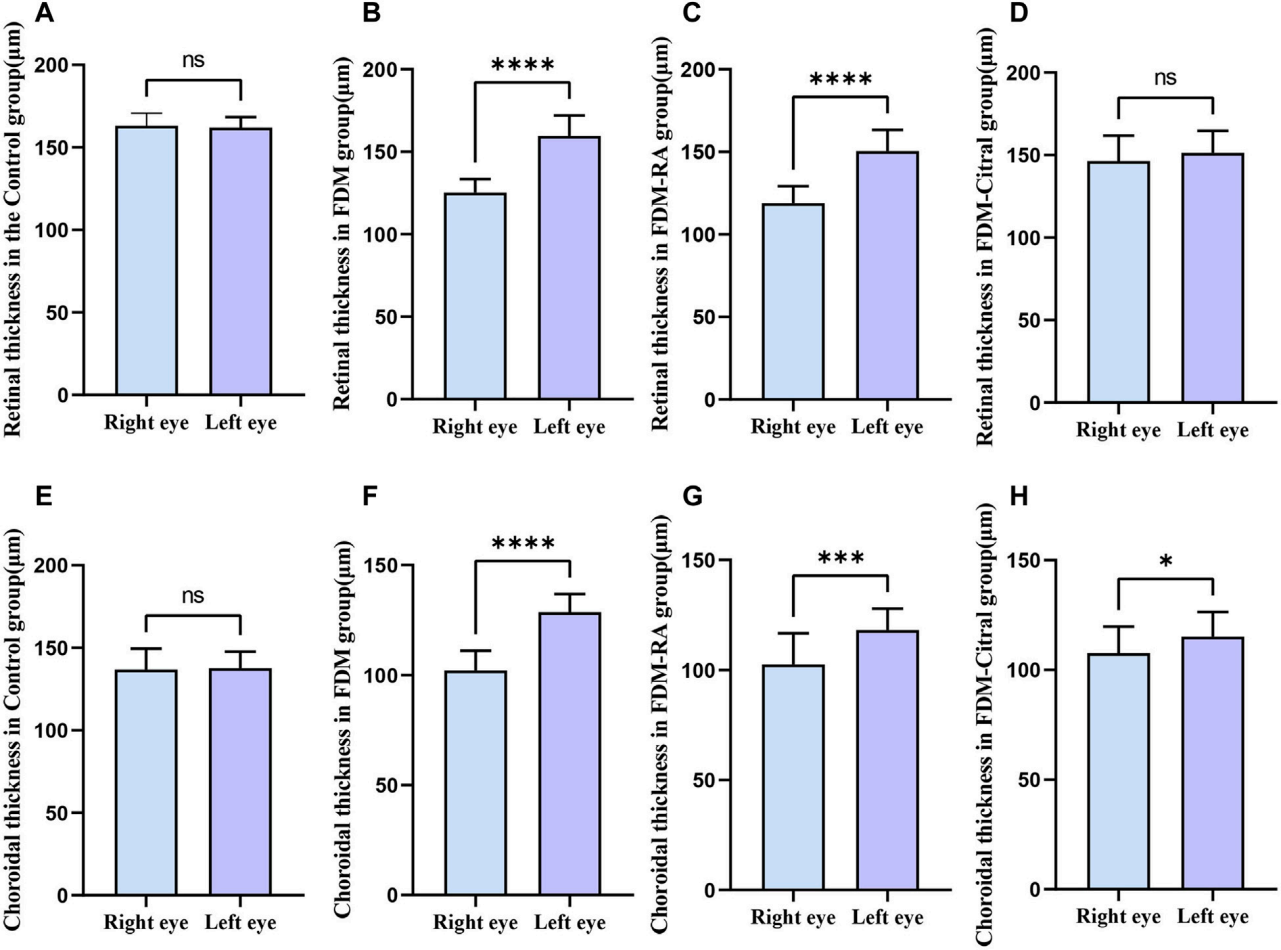
Comparison of RT and CT between the left and right eyes in each group (μm). Note. **(A)**, Comparison of RT between left and right eyes in Control group; **(B)**, Comparison of RT between left and right eyes in FDM group; **(C)**, Comparison of RT between left and right eyes in FDM + RA group; **(D)**, Comparison of RT between left and right eyes in FDM + Citral group; **(E)**, Comparison of CT between left and right eyes in Control group; **(F)**, Comparison of CT between left and right eyes in FDM group; **(G)**, Comparison of CT between left and right eyes in FDM + RA group; **(H)**, Comparison of CT between left and right eyes in FDM + Citral group; ns means that the difference is not statistically significant; *Indicates *p* ≤ 0.05; ** Indicates *p* ≤ 0.01; *** Indicates *p* ≤ 0.001; **** Indicates *p* ≤ 0.0001.

There was a statistical difference between FDM group and FDM + RA group in RT of left and right eyes, and RT of right eye was significantly less than that of left eye (Figures 8B, C). There was statistically significant difference between the left and right eye CT of FDM group and FDM + RA group (Figures 8F, G). In FDM + Citral group, there was statistical difference in CT between left and right eyes, and the CT of right eye was significantly smaller than that of left eye (Figure 8H), but not in RT (Figure 8D). There was no difference in CT and RT between the left and right eyes between Control groups (Figures 8A, E).

## Discussion

In this study, we discussed the effect of RA on the intraocular parameters of FDM guinea pigs, and, in particular, analyzed the RT and CT measured by EDI-OCT in guinea pigs under different induction factors. We found that the eye axis and refractive degree of young guinea pigs after form deprivation increased. The AL of the right eye was 0.57 ± 0.05 mm larger than that of the control eye and the right eye was myopia (−2.68 ± 0.26 D), whereas the left eye was still hyperopia (2.14 ± 0.44 D). However, the retina and choroid did decrease correspondingly (RT in the right eye was 34.38 ± 3.24 μm smaller than that in the left eye, whereas CT was 26.48 ± 2.67 μm smaller). Moreover, the degree of myopia of FDM guinea pigs treated with RA was more serious, with RE reaching −4.84 ± 0.59 D and AL 8.46 ± 0.21 mm; the RT of the right eye in the FDM + RA group was 118.90 ± 10.30 μm, which was smaller than that in the FDM group (125.30 ± 8.13 μm), although the CT results of the two groups were almost the same (102.10 ± 9.01 μm in FDM group, 102.60 ± 14.10 μm in FDM + RA group). However, the myopia trend seemed to be alleviated by RA inhibitors. The RE was −1.85 ± 0.56 D, AL was 7.88 ± 0.17 mm, and RT was 146.50 ± 15.29 μm, however, this did not seem to affect the choroid, its CT was 107.60 ± 12.13 μm, which is far less than 136.90 ± 12.61 μm of the control group. Furthermore, we found that RA might aggravate the thinning tendency of the retina in FDM guinea pigs through the results of EDI-OCT. Although there was no statistically significant difference between the RT and CT results of the right eye in the FDM and FDM + RA groups, the RT in the FDM + RA group was the smallest among the four groups, and the RT in the FDM + Citral group was significantly greater than that in the FDM and FDM + RA groups, indicating that RA might aggravate the retinal thinning trend in FDM. This trend was suppressed with RA antagonists. However, RA seemed to have no effect on the change of CT in FDM guinea pigs (there was no statistical difference in CT among the FDM, FDM + RA, and FDM + Citral groups). Also, we found that the IOP of FDM guinea pigs after RA treatment increased and IOP was normal after citral treatment.

RA is present widely in retina and choroid, which is considered to be a key signaling molecule regulating eye growth and might be associated with myopia (Mertz and Wallman, 2000). RA is regulated mainly by retinalaldehyde dehydrogenase 1 and 2 (RALDH1 and RALDH2), which are present in the retina and choroid. Harper et al. found that these two enzymes exist in the choroid and retina of human eyes, especially in the choroid (Harper et al., 2015). However, only RALDH2 changes during the recovery of the experimental myopia model. Summers et al. reported the changes of RALDH2^+^ cells in the choroid of the chick myopia model during the recovery stage of myopia, finding that the RALDH2^+^ cells existed mainly in the choroid stroma and vascular attachment, and would continue to rise during the recovery stage of myopia. The choroid would also thicken within 4 days of the recovery stage and the trend of RE, AL, and CT increasing was consistent with the change of RALDH2 (Summers et al., 2020). In addition, the phenomenon of CT enlargement in the myopic model has also been reported by Liang et al. who found that the retina and choroid of chicks would thicken within 6 days of form deprivation, and then the thickness of the retina would first return to normal. They found that the concentration of sodium and chlorine in the retina and choroid of myopic eyes was lower than that of normal eyes, resulting in tissue edema and increased thickness (Liang et al., 2004). Our study found that the retina of FDM guinea pigs may be thickened after 4 weeks of RA induction, but the choroid has no obvious change, and the CT results of FDM + RA group were even larger than those of FDM guinea pigs without RA induction (CT of the FDM group: 102.10 ± 9.01 μm; CT of the FDM + RA group: 102.60 ± 14.10 μm). We speculated that this might be related to the stage of myopia that the choroid and retina edema occurred during, but the retina first recovered and became thinner with the aggravation of myopia. However, it is not clear whether our phenomenon is related to the mediation of related proteins as other studies have shown.

In fact, in both FDM and LIM models of guinea pigs and chickens, it has been confirmed that all-trans RA (atRA) will increase (Bitzer et al., 2000; Huang et al., 2011), whereas, in hyperopia models, it will decrease, although the specific mechanism of RA regulating refractive changes is not clear. Also, the significant increase of RALDH2 protein production in the choroid, and the change of its content in the retina might be related closely to the formation of myopia. In the LIM model, guinea pigs developed myopia tendency after wearing a −6 D lens for only 2 weeks, and the content of RA and the production of RALDH2 protein also increased. However, in guinea pigs with myopia recovery, the protein was reduced in the retina, but not in the choroid, which is contrary to the results in chicken mentioned earlier (this might be related to the different myopia mechanisms between chickens and guinea pigs). This study suggests that RA in the retina and choroid is involved in the regulation of LIM guinea pigs (Mao et al., 2012). Nevertheless, although both LIM and FDM models form axial myopia, their mechanisms are not completely the same, and the mechanism of RA in FDM guinea pigs might also be different from other types of animal myopia models.

The FDM guinea pig model was selected for our study, which is a commonly used myopia model because of their docile temperament and similar eyeball development to humans (Cheng et al., 2014; Cheng et al., 2015). Studies have reported that the structural changes to the retina of FDM guinea pigs were observed by light and electron microscopy. The depth of the vitreous cavity, retina, and sclera of the RE and AL were thinned after form deprivation, and the activity of superoxide dismutase (SOD) in FDM eyes was reduced significantly. They believed that oxygen free radicals might be related to the formation of FDM (Zi et al., 2020). The results of their study are similar to the changes of intraocular parameters in our FDM group. In addition, we can see that the RE, AL of FDM eyes in guinea pigs are increased compared with those in the FDM group after RA induction. Although the difference of RT comparison results was not statistically significant, there was a decreasing trend. Furthermore, in contrast to the earlier research, we used OCT technology to collect the structure of the retina and choroid of living guinea pigs, which can improve the efficiency of experiments. EDI-OCT can be used clinically to measure CT and RT results of patients (Park et al., 2013). However, animals have poor compliance compared with humans because it is difficult for animals to cooperate closely with instruments for measurement. In addition, eyeballs are often smaller in animals, which is one of the reasons for the difficulty in obtaining intraocular parameters of small animals. Based on the docile characteristics of guinea pigs, in this study, our guinea pigs were awake for OCT scanning, and we also obtained retinal and choroid images successfully, reducing the mortality from anesthesia.

Additionally, we found that the IOP of FDM guinea pigs after RA increased, which has never been reported before. However, myopia is related to glaucoma, and HM is an independent risk factor for glaucoma (Jonas et al., 2020), and, the higher the degree of myopia, the higher the risk of glaucoma. In our study, guinea pigs in the FDM + RA group had the highest degree of myopia in the right eye. We speculated that this might be the reason for the high IOP in this group, although it might also be related to the pharmacological effects of RA.

It should be mentioned that our research has the following limitations. 1. We have not discussed the changes of choroidal blood flow in depth. Optical coherence tomography angiography (OCTA) can determine changes of choroidal blood flow and vascular density, whereas the degree of myopia is correlated negatively with the density of choroidal choriochorionic capillaries (Liu et al., 2021; Li et al., 2022). Unfortunately, our laboratory does not have OCTA instruments. 2. We analyzed the intraocular parameters of RA acting on FDM guinea pigs, but did not further study the pharmacological mechanism. In future research, we will strive to address these limitations.

## Conclusion

Morphological deprivation in guinea pigs results in thinning of the retina and choroid. Exogenous RA can aggravate the tendency of myopia in FDM guinea pigs. Meanwhile, exogenous RA can cause an increase of IOP in FDM guinea pigs. However, after RA inhibition, the refractive state and AL of FDM guinea pigs were reduced. At the same time, RA might aggravate retinal thinning in FDM guinea pigs, although it seems to have no obvious effect on choroidal thinning. The study of RA might provide an important breakthrough in understanding the mechanism of myopia.

## Data Availability

The original contributions presented in the study are included in the article/Supplementary Material, further inquiries can be directed to the corresponding authors.
